# Keokuk County Medical Society

**Published:** 1858-06

**Authors:** W. B. Smith


*Proceedings of the Keokuk County, Iowa, Medical Association. Reported by W. B. Smith, M. D.*

The physicians of Keokuk county met at Sigourney, May 13th, for the purpose of re-organizing the Keokuk County Medical Association, Dr. W. B. Smith was called to the Chair, and Dr. H. W. Jay appointed Secretary.

Election of Officers.—After the transaction of some miscellaneous business, the Association proceeded to elect officers for the ensuing year with the following result:

President, Dr. A. Parks; Vice President, Dr. F. A. Dorr; Recording Secretary, Dr. H. W. Jay; Corresponding Secretary, Dr. W. B. Smith; Treasurer, Dr. A. C. Price; Board of Censors, Drs. Belden, Smith, Parks, Dorr, and Price.

Several gentlemen were appointed to read essays at the next regular meeting.

Delegates.—Drs. A. Parks, F. A. Dorr, H. W. Jay, and W. B. Smith were appointed to represent this Association in the Iowa State Medical Association.

On motion, the code of ethics of the American Medical Association was adopted.

